# Robust Artificial Interlayer with High Ionic Conductivity and Mechanical Strength toward Long‐Life Na‐Metal Batteries

**DOI:** 10.1002/smsc.202300038

**Published:** 2023-06-07

**Authors:** Xianming Xia, Kaizhi Chen, Shitan Xu, Yu Yao, Lin Liu, Chen Xu, Xianhong Rui, Yan Yu

**Affiliations:** ^1^ Guangdong Provincial Key Laboratory on Functional Soft Condensed Matter School of Materials and Energy Guangdong University of Technology Guangzhou 510006 China; ^2^ Hefei National Research Center for Physical Sciences at the Microscale Department of Materials Science and Engineering CAS Key Laboratory of Materials for Energy Conversion University of Science and Technology of China Hefei Anhui 230026 China; ^3^ Academy for Advanced Interdisciplinary Studies Southern University of Science and Technology Shenzhen 518055 China

**Keywords:** artificial interlayer, high mechanical toughness, interface engineering, long cycling life, sodium-metal anodes

## Abstract

Sodium metal, benefiting from its high theoretical capacity and natural abundance, is regarded as a promising anode for sodium‐metal batteries (SMBs). Unfortunately, the uncontrollable sodium dendrites formation caused from the sluggish ion‐transport kinetics and fragile solid electrolyte interphase (SEI) interlayer induces a low Coulombic efficiency and poor cycling stability. Constructing an artificial SEI interlayer with high ionic conductivity, stability, and mechanical toughness is an effective strategy for Na‐metal anode, yet it still presents major challenge for high current density and long cycling life. Herein, an artificial SEI interlayer composed of Na–Sn alloy, Sn, and Na_2_Te (denoted as NST) is designed via in‐situ conversion/alloying reaction of tin telluride (SnTe) with Na. Such artificial interlayer possesses rapid Na^+^‐transport kinetics and high Young's modulus (5.3 GPa), benefitting to even Na plating/stripping and suppressing Na dendrite growth. Owing to these merits, the symmetrical Na/NST cell presents an ultralong cycle life span over 1390 h with a small voltage hysteresis at 1 mA cm^−2^ with 1 mAh cm^−2^. And the Na_3_V_2_(PO_4_)_3_ (NVP)||Na/NST full cell exhibits a prolonged life of 1000 cycles with a high‐capacity retention of 88% at 5C. Herein, a promising strategy is provided to construct a high‐performance artificial interlayer for SMBs.

## Graphical abstract

An artificial solid electrolyte interphase layer composed of Na–Sn alloy, Sn, and Na_2_Te is designed to protect the Na anode via in situ conversion/alloying reactions. It presents rapid ionic conductivity, high Young's modulus, and structure stability, achieving homogeneous Na deposition with dendrite‐free morphology and ultralong life span over 1390 h at 1 mA cm^−2^/1 mAh cm^−2^.

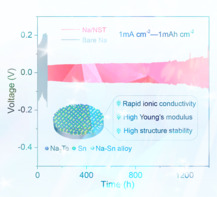

## 1 Introduction

Lithium batteries have been widely employed in various fields.^[^ ^1^, ^2^, ^3^, ^4^, ^5^, ^6^, ^7^ ^]^ However, the limited resource of lithium impedes their large‐scale application.^[^ ^8^, ^9^, ^10^, ^11^, ^12^, ^13^ ^]^ Therefore, it is urgent to seek a cost‐effective and more gainable energy‐storage systems. Compared to lithium batteries, sodium (Na) batteries present a lot of advantages, including the cost‐effective and plenty of the raw materials.^[^ ^14^, ^15^, ^16^, ^17^ ^]^ Among the various anode candidates for Na batteries, the Na metal anode is considered as the eventual material, owing to its high theoretical capacity and low redox potential.^[^ ^18^, ^19^, ^20^, ^21^ ^]^ However, the metallic Na with high reactivity can react with the electrolyte to form a solid electrolyte interphase (SEI) on the Na surface.^[^ ^22^, ^23^, ^24^, ^25^ ^]^ This SEI interlayer can mitigate the further side reaction and realize the ionic transportation. Unfortunately, the naturally formed SEI usually presents nonuniform structure and fragile character, which induce inhomogeneous Na deposition and poor electrochemical performance.

To tackle these challenges, some efforts have been devoted for high‐performance Na‐metal batteries (SMBs).^[^ ^26^, ^27^, ^28^, ^29^, ^30^, ^31^ ^]^ It is well known that the composition of natural SEI is critically related to the additive, solvent, and salt of the electrolytes.^[^ ^32^, ^33^, ^34^, ^35^ ^]^ According to the previous reports, the naturally formed SEI usually consists of dual layer in common carbonate‐based electrolytes, where the inner layer and outer part are inorganic and organic phases, respectively.^[^ ^23^, ^36^ ^]^ However, the organic layer is easily dissolved during the continued cycling process,^[^ ^37^ ^]^ while the inorganic‐rich SEI displays impermeability to the electrolyte solvent. Electrolyte engineering via adding additives to construct an inorganic‐rich SEI is an effectively method, compared to the solvent and salt regulation. For instance, Zheng et al. designed a NaF‐rich inorganic SEI interlayer via introducing the sodium hexafluoroarsenate additive.^[^ ^38^ ^]^ However, the continuous consumption of the electrolyte additives during the plating/stripping process induces poor cycling stability. Recently, constructing an artificial SEI interlayer for SMBs via pretreatment has attracted researchers’ efforts due to its simpleness and effectiveness. For instance, Tian et al. fabricated an artificial SEI interlayer of NaI on Na via 2‐iodopropane pretreatment.^[^ ^39^ ^]^ The obtained artificial SEI interlayers display rapid ionic transport kinetics, realizing a homogeneous Na deposition and no Na dendrites. Unsatisfactorily, the electrodes modified by artificial SEI interlayer usually exhibit poor cycling life (<800 h) in carbonate electrolytes (vital for practical applications) and high‐voltage hysteresis.^[^ ^39^, ^40^, ^41^, ^42^ ^]^ Therefore, it is imperative to explore a stable and robust artificial SEI interlayer for long‐term cycling SMBs.

Herein, we designed an artificial SEI interlayer with superior ionic conductivity and outstanding mechanical toughness for SMBs via a simple and promising SnTe pretreatment method. The artificial SEI interlayer composed of Na–Sn alloy, Sn, and Na_2_Te presents a reduced activation energy barrier (40.9 kJ mol^−1^) and high Young's modulus (5.3 GPa), which can promote rapid diffusion of the Na^+^, realizing homogeneous Na deposition. And the high Young's modulus of the artificial SEI interlayer mitigates the Na dendrites growth effectively during the plating/stripping cycling. All these merits of the artificial SEI interlayer endow the batteries with a low dynamic barrier and excellent cycling stability. Therefore, the symmetrical cell assembled with the artificial interlayer‐modified Na electrode (denoted as Na/NST) presents a superior cycle stability over 1390 h at 1 mA cm^−2^/1 mAh cm^−2^ in carbonate‐based electrolyte, compared to the symmetrical bare Na cell (80 h). The full cell assembled by the commercial Na_3_V_2_(PO_4_)_3_ (NVP) cathode and the Na/NST anode (NVP||Na/NST) demonstrates an outstanding capacity of 65 mAh g^−1^ even at 50C. Moreover, the NVP||Na/NST full cell delivers an excellent cycling stability with an ultrahigh reversible discharge capacity of 90 mAh g^−1^ even after 1000 cycles at 5C.

## 2 Results and Discussion

**Figure 1:**
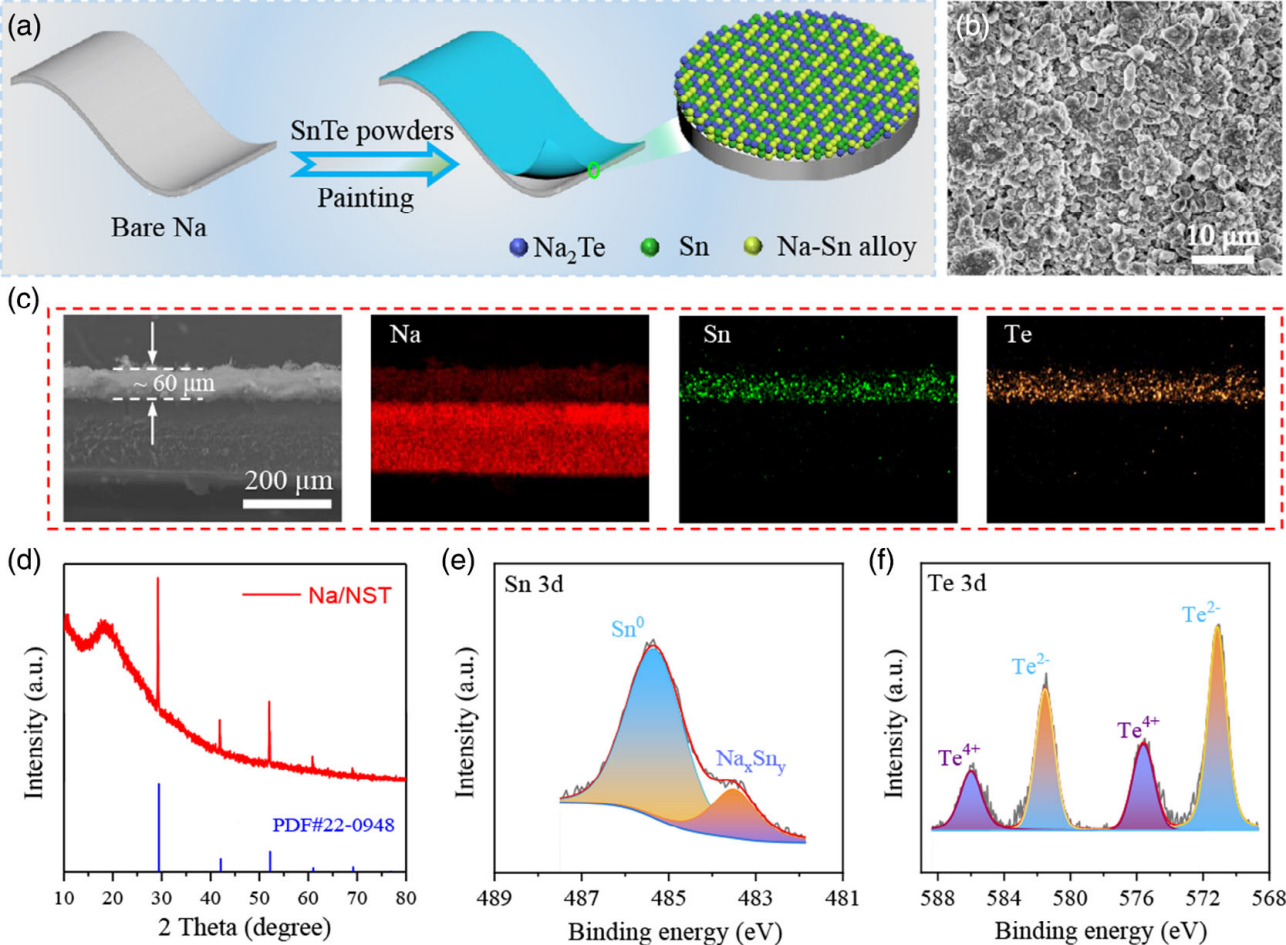
a) Schematic illustration of the fabrication procedure of the Na/NST. b) SEM images of the Na/NST. c) Cross‐sectional SEM image and EDS elemental mapping of Na/NST. d) X‐ray diffraction pattern of the Na/NST. e,f) High‐resolution XPS spectra of Sn 3d (e) and Te 3d (f) for the Na/NST.

The fabrication process of Na/NST anode is illustrated in **Figure** **1**a. In this process, the tin telluride (SnTe) powders (Figure S1, Supporting Information) were painted onto the homemade Na metal foil. It is notable that the whole fabrication process of the electrodes was carried out in an Ar‐filled glove box. After painting the SnTe powders, the inherent silvery color of metallic Na changed to the black gray (Figure S2, Supporting Information), indicating the formation of the artificial interlayer.

The morphology of the artificial interlayer was investigated via scanning electron microscopy (SEM). The SEM images (Figures 1b and S3, Supporting Information) reveal that the artificial interlayer is consisted of compactly attached particles. Moreover, the SEM–energy‐dispersive spectroscopy (EDS) element mapping displays the even distribution of Na, Sn, and Te elements in a large zone (Figure S4, Supporting Information), which indicates that the artificial interlayer densely and evenly covers on the Na substrate. Meanwhile, the cross‐sectional image of the SEM also confirms that the artificial interlayer compactly covers on the Na substrate and the thickness of the artificial interlayer is ≈60 μm (Figure 1c). Moreover, to examine the composition of the artificial interlayer, various characterization techniques have been conducted. As shown in the Figure 1d, from X‐ray diffraction patterns, the obvious crystalline phase structure of raw SnTe (PDF No. 46‐1210) is not detected, which indicates that the SnTe has been reacted with the Na metal to generate fine low‐crystalline (or amorphous) compounds. And a broad peak located at around 19° can be observed, which is assigned to the polyimide tape that covered on Na/NST for testing protection (Figure S5, Supporting Information). In the high‐resolution Sn 3d spectrum obtained from X‐ray photoelectron spectroscopy (XPS), two peaks located at 485.4 and 483.5 eV correspond to the metallic Sn and Na_*x*_Sn_*y*_ alloy, respectively (Figure 1e), implying that the Sn^2+^ can be reduced to metallic Sn, and then alloy with highly redox‐active metallic Na.^[^ ^43^, ^44^ ^]^ And as shown in Figure 1f, two paired peaks can be detected in the high‐resolution Te 3d spectrum. And, the strong peaks located at 571.2 and 581.5 eV are assigned to 3d_5/2_ and 3d_3/2_ of Te^2−^, respectively, indicating the formation of Na_2_Te. Moreover, the minor peaks located at 575.6 and 586.0 eV are pertained to Te^4+^, which are ascribed to the slight surface oxidation during the sample transfer process, and this phenomenon is commonly observed in the XPS spectra of Na_2_Te.^[^ ^45^, ^46^ ^]^

**Figure 2:**
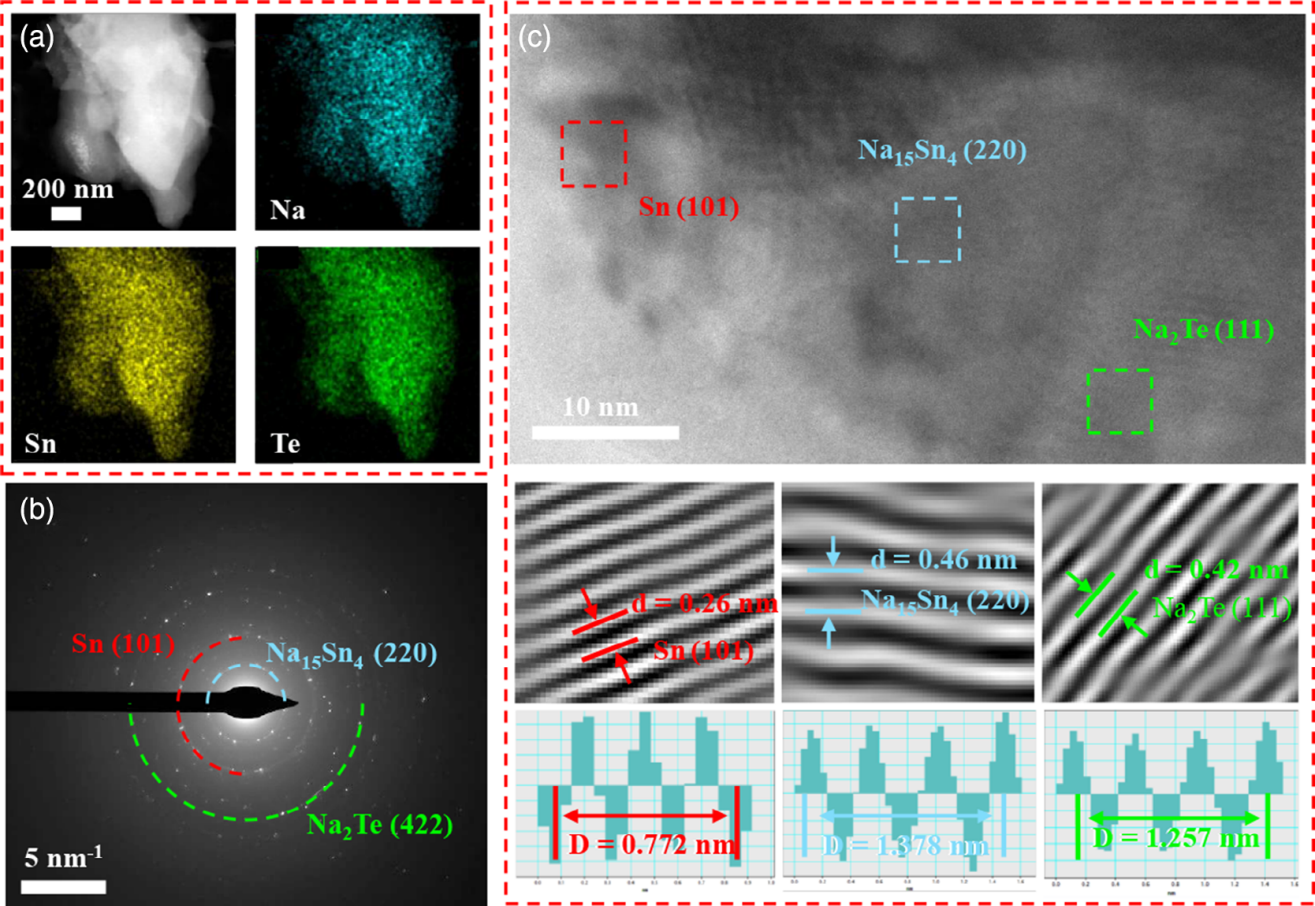
a) Cryogenic electron energy loss spectroscopy images of the particles of the artificial NST SEI interlayer. b) SAED pattern and c) high‐resolution CryoTEM image and the corresponding inverse fast Fourier transform images of the artificial NST interlayer.

The microstructure of the artificial NST interlayer was also investigated via the advanced cryogenic transmission electron microscopy (Cryo‐TEM) technique. The Cryo‐TEM image (Figure S6, Supporting Information) further proves the formation of the serried particles after the in situ conversion/alloy reactions. As displayed in the **Figure** **2**a, the cryogenic electron energy loss spectroscopy images of the particles of artificial interlayer exhibit that the Na, Sn, and Te elements are uniformly distributed in the artificial interlayer, suggesting the thorough reaction between SnTe and metallic Na. The selected area electron diffraction pattern (SAED) in Figure 2b reveals different diffraction rings of Na_15_Sn_4_ (220), Sn (101), and Na_2_Te (422) planes. They are further confirmed by high‐resolution Cryo‐TEM observation of the artificial interlayer (Figure 2c), and as revealed by inverse fast‐Fourier‐transform analysis, the interplanar spacing of 0.46, 0.42, and 0.26 nm corresponds to the Na_15_Sn_4_ (220), Na_2_Te (111), and Sn (101), respectively. These results consistently affirm the successful fabrication of artificial hybrid interlayer consisted of Sn, Na–Sn alloy, and Na_2_Te. For comparison, the Na/Te and Na/Sn electrodes were also constructed via the similar pretreatment method of painting Te and Sn powders on Na metal (Figure S7, Supporting Information).

**Figure 3:**
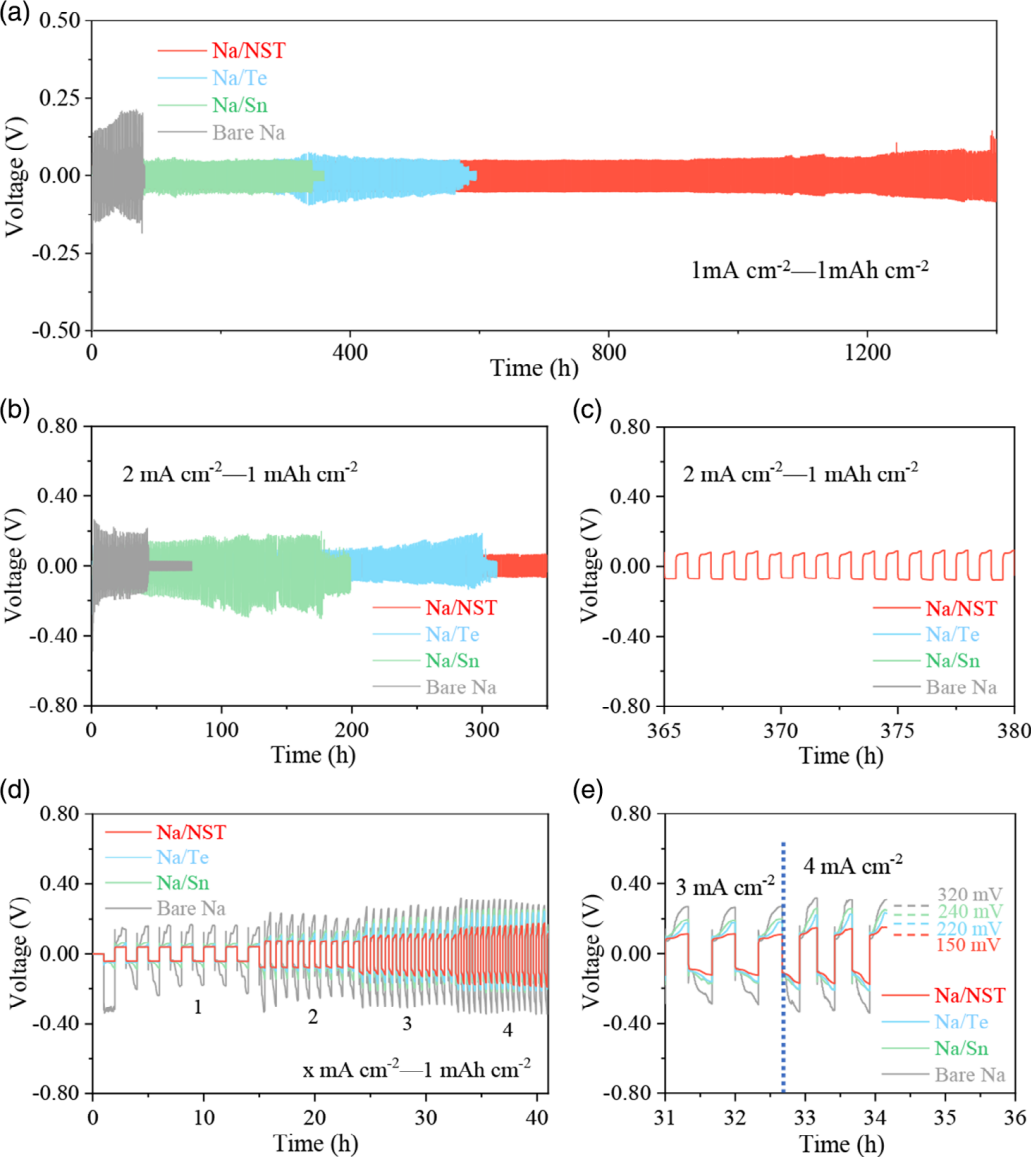
Electrochemical performance of the symmetrical cells based on bare Na, Na/Sn, Na/Te, and Na/NST electrodes. a,b) Cycling performance of the symmetrical cells at 1 mA cm^−2^ (a) and 2 mA cm^−2^ (b). c) Detailed plating/stripping curves of the symmetrical cells at 2 mA cm^−2^ from 365 to 380 h. d) Rate performance from 1 to 4 mA cm^−2^ and e) overpotentials at high current densities of the symmetrical cells.

The validity of the artificial NST interlayer for protecting the Na‐metal anode was investigated via galvanostatic charge–discharge test of symmetrical cells in carbonate‐based electrolyte. **Figure** **3**a presents the plating/stripping stability of the symmetrical cells. The symmetrical bare Na cell displays a short cycling life span (80 h, Figure S8a, Supporting Information) with a large overpotential at 1 mA cm^−2^ with 1 mAh cm^−2^, which causes severe Na dendrite growth, resulting in short circuit. However, the electrode modified by various artificial interlayer presents a prolonged cycling life span. For instance, the symmetrical Na/Sn and Na/Te cells can stably cycle over 340 and 580 h, respectively (Figure S8b,c, Supporting Information). Conspicuously, the symmetrical Na/NST cell displays an excellent cycling stability over 1390 h and still retains a stable and smooth voltage profile (Figure S8d, Supporting Information). As the current density changes from 1 to 2 mA cm^−2^, the symmetrical bare Na cell presents an increased overpotential and apparent voltage fluctuation (Figure 3b), and it can be observed that the voltage curve quickly drops after 43 h (Figure S9a, Supporting Information), suggesting the accelerated and uncontrolled Na dendrite growth under high current density. The symmetrical Na/Sn and Na/Te cells exhibit reduced voltage polarization. However, the fluctuant voltage behavior can be detected after 170 and 300 h in the symmetrical Na/Sn and Na/Te cells (Figure S9b,c, Supporting Information), respectively, implying unstable Na plating/stripping behavior. In contrast, the symmetrical Na/NST cell presents prolonged cycle stability of around 380 h with stable and smooth voltage profile (Figure 3c), suggesting the excellent stability with the effect from the artificial SEI interlayer. Remarkably, compared to the previous reported Na‐metal anodes, our Na/NST also exhibits a superior cycling life span, as shown in Table S1 (Supporting Information).

To further reveal the advantages of the Na/NST electrode, the rate performance of the symmetrical cells was also compared. The symmetrical bare Na cell always presents the highest voltage hysteresis at the various current densities (from 1 to 4 mA cm^−2^) (Figure 3d). However, the symmetrical Na/Sn and Na/Te cells display a significantly reduced voltage hysteresis. Moreover, the symmetrical Na/NST cell displays the best rate performance, possessing the lowest overpotential in the entire rate process. Specially, the symmetrical Na/NST cell merely shows a lower overpotential of 150 mV at the high current density of 4 mA cm^−2^, compared to the symmetrical Na/Te (220 mV), Na/Sn (240 mV), and bare Na (320 mV) cells (Figure 3e). These electrochemical performances reveal that the artificial interlayer with a composition of Sn, Na–Sn alloy, and Na_2_Te does not only prolong the cycling, but also improve the Na^+^‐transport kinetics for a superior rate behavior.

**Figure 4:**
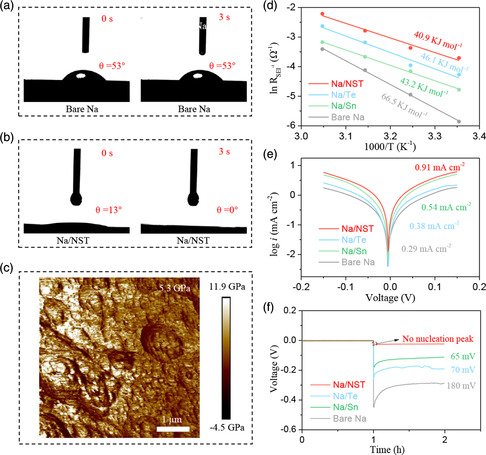
a,b) Wettability of the carbonate‐based electrolytes on the bare Na (a) and the Na/NST (b) electrode surface. c) The Young's modulus distribution of the Na/NST electrode. d) Activation energy of the Na/NST, Na/Sn, Na/Te, and bare Na electrodes for Na‐ion diffusion. e) The exchange current density of the bare Na, Na/Sn, Na/Te, and Na/NST electrode. f) The nucleation overpotential of bare Na, Na/Sn, Na/Te, and Na/NST electrodes.

To understand the effect of the artificial interlayer on electrochemical performance, the underlying mechanism was investigated. As presented in **Figure** **4**a,b, the wettability of the carbonate‐based electrolytes on both the bare Na and Na/NST electrodes was tested. A high contact angle of 53° on the bare Na electrode can be observed. And even the dropping time reaches 3 s, the electrolyte cannot spread out sustaining a contact angle of 53° (Figure 4a), indicating a poor wettability on the bare Na electrode. On the contrary, a great wettability can be detected on the Na/NST electrode, that the electrolyte droplet completely penetrates into the Na/NST electrode after 3 s (Figure 4b). The superior wettability is beneficial for the reducing interfacial resistance and uniform distribution of the Na^+^, realizing homogenous Na deposition. The mechanical strength of the artificial SEI interlayer was studied via atomic force microscope. The electrode modified by SnTe exhibits a high Young's modulus of 5.3 GPa (Figure 4c), which is higher than that of the previous reports of bare Na.^[^ ^47^, ^48^ ^]^ Therefore, the Na/NST electrode can suppress the growth of Na dendrite, benefitting to homogeneous Na deposition and prolong the plating/stripping cycling.

The rapid Na^+^ diffusion in the SEI interlayer is a critical factor for homogenous Na deposition and superior electrochemical performance. And the activation energy (*E*_a_) is an importance parameter to explore the Na‐ion diffusion capacity in SEI. The electrochemical impedance spectroscopy (EIS) was tested at different temperatures ranging from 25 to 55 °C, as exhibited in Figure S10 (Supporting Information). According to the Arrhenius equation,^[^ ^23^, ^49^ ^]^ a high *E*_a_ value of 66.5 kJ mol^−1^ is obtained in symmetrical bare Na cell (Figure 4d), implying the high kinetic barriers for Na^+^ diffusion in the native SEI interlayer, which will induce the nonuniform Na^+^ deposition and formation of the Na dendrites. Lower *E*_a_ values of 43.2 and 46.1 kJ mol^−1^ are obtained in symmetrical Na/Sn and Na/Te cell, respectively. Moreover, the lowest *E*_a_ value of 40.9 kJ mol^−1^ is achieved in symmetrical Na/NST cell, indicating that the electrode modified by SnTe can promote fast Na‐ion diffusion. The superior dynamics of the Na/NST electrode is further evidenced by the exchange current density. As displayed in Figure 4e, compared to the Na/Te (0.38 mA cm^−2^), Na/Sn (0.54 mA cm^−2^), and bare Na (0.29 mA cm^−2^), the Na/NST processes a higher exchange current density of 0.91 mA cm^−2^, suggesting the rapid Na‐ion diffusion, and achieving homogeneous Na deposition. Moreover, according to the previous reports, the theoretical simulation reveals that the Na–Sn alloy and Na_2_Te present excellent sodiophilicity and Na^+^ diffusion kinetics, which promote the fast Na^+^ adsorption and diffusion, realizing homogeneous Na deposition.^[^ ^50^, ^51^ ^]^ Furthermore, the improved dynamics for Na^+^ adsorption and diffusion are further evidenced via testing the nucleation overpotentials. As presented in Figure 4f, the bare Na electrode exhibits a higher nucleation overpotentials of 180 mV, compared to the Na/Te (70 mV) and Na/Sn (65 mV) electrodes. However, no evident nucleation peak is observed in the Na/NST electrode, implying the high sodiophilicity and enormously promoted Na‐ion‐transport kinetics.

**Figure 5:**
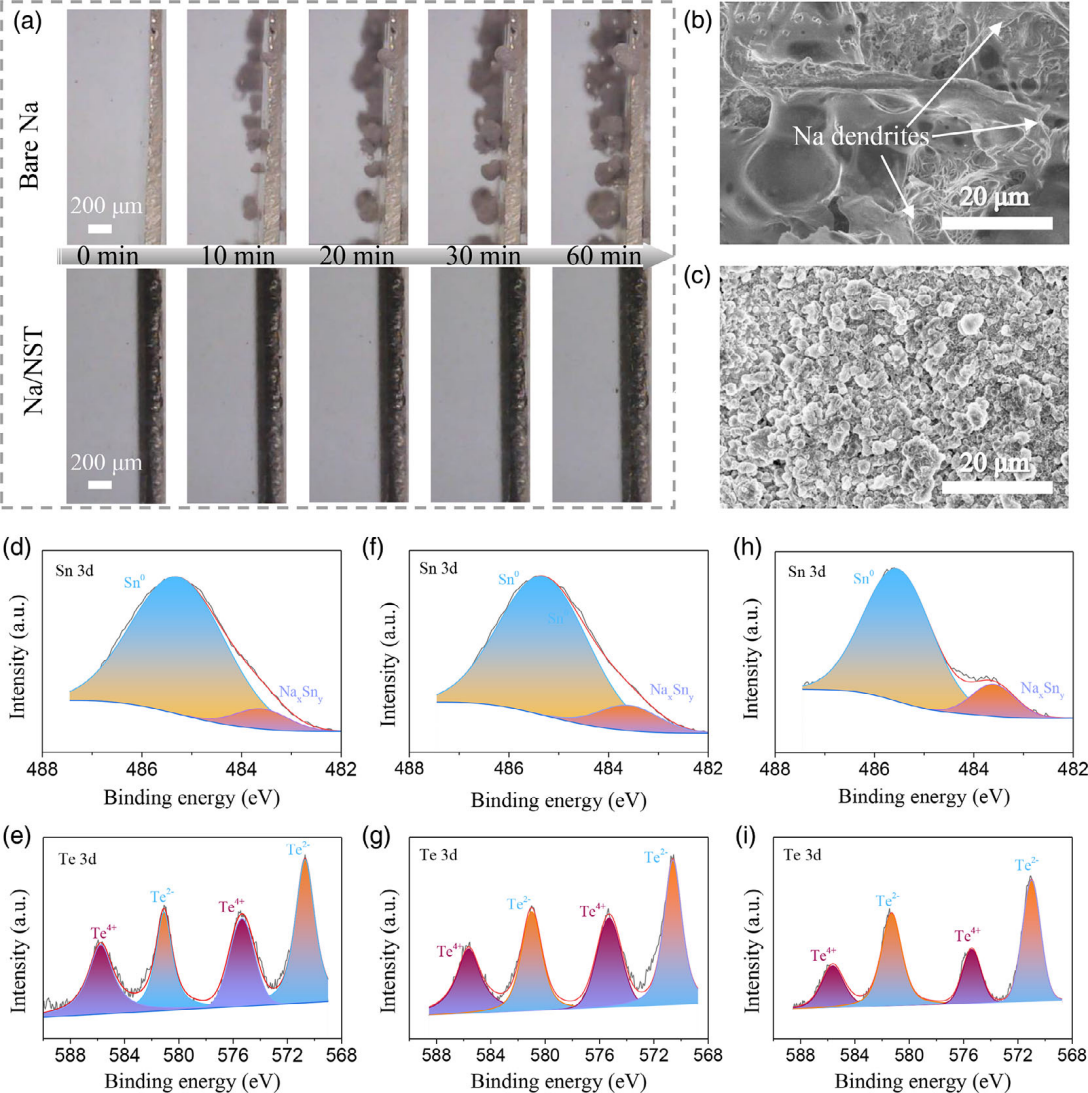
a) Investigation of Na plating behavior on different electrodes. b,c) SEM images of electrode surface of bare Na (b) and Na/NST (c) after 10 cycles. d–i) high‐resolution XPS spectrum of Na/NST electrodes after plating 1 mAh cm^−2^ (d,e), 2 cycles (f,g), and 10 cycles (h,i) at 1 mA cm^−2^ with 1 mAh cm^−2^.

To further investigate the effects of the artificial interlayer for suppressing Na dendrite growth, the Na plating process is directly observed via transparent quartz symmetrical cell at 1 mA cm^−2^. As displayed in **Figure** **5**a, both the Na/NST and bare Na electrodes display a flat surface before the plating process. However, mossy‐like Na can be discovered on the bare Na electrode after plating for only 10 min. And the mossy‐like Na totally covers the bare Na electrode when plating for 60 min, revealing the cause of the poor cycling performance of the bare Na electrode. By contrast, the smooth surface can be still retained on the Na/NST electrode, and no mossy‐like Na can be observed even after plating for 60 min, which clearly indicates that the Na/NST electrode can significantly mitigate the Na dendrite formation, benefitting to the stable plating/stripping process for prolonged cycling life. Moreover, the optical picture shows that the initial metal luster of the bare Na electrode disappears after cycling (Figure S11a, Supporting Information), suggesting masses of dead Na or Na dendrite covering the electrode surface. However, no obvious change can be detected on the Na/NST electrode (Figure S11b, Supporting Information), indicating the high stability of the artificial interlayer and homogeneous Na plating/stripping cycling. Furthermore, the micromorphology of these electrodes was also investigated via SEM after cycling at 1 mA cm^−2^ with 1 mAh cm^−2^. It can be observed that there are plenty of the dead Na and dendrite‐like Na on the bare Na electrode (Figures 5b and S12a, Supporting Information), which is resulted from the nonuniform Na plating/stripping cycling. On the contrary, a smooth surface can be still presented on Na/NST electrode, and dendrite‐free morphology after 10 cycles (Figures 5c and S12b, Supporting Information), confirming the homogeneous Na stripping/plating cycling. Additionally, the stability of the interlayer is critically important for long‐term cycling and was investigated. As shown in Figure 5d,e, the composition of the Sn, Na_*x*_Sn_*y*_ alloy, and Na_2_Te can be detected in the high‐resolution Sn 3d (Figure 5d) and Te 3d (Figure 5e) after plating 1 mAh cm^−2^, respectively. Moreover, after plating/stripping for 2 cycles at 1 mA cm^−2^ with 1 mAh cm^−2^, the high‐resolution Sn 3d (Figure 5f) and Te 3d (Figure 5g) of Na/NST electrode reveal that the retention of Sn, Na_*x*_Sn_*y*_ alloy, and the Na_2_Te in the artificial interlayer, suggesting the high stability of the Na/NST electrode. To further confirm this result, the XPS spectra of Na/NST electrode after 10 cycles were also investigated. As displayed in Figure 5h, the two peaks located at 483.5 and 485.4 eV correspond to the Na_*x*_Sn_*y*_ alloy and Sn, respectively. And the peaks at 571.2 and 581.5 eV in the high‐resolution Te 3d (Figure 5i) are pertained to Na_2_Te. Therefore, it can be concluded that the stable Na/NST electrode with the functional artificial interlayer endows rapid ion diffusion kinetics for uniform Na plating and suppresses dendrite growth, realizing prolonged cycling life span during the stripping/plating process.

**Figure 6:**
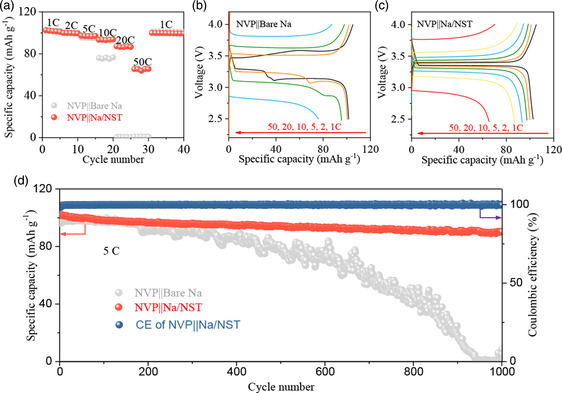
a) Rate performance of the Na_3_V_2_(PO_4_)_3_ (NVP)||Na/NST and NVP||bare Na full cells. b,c) Charge–discharge curve of the NVP||bare Na full cells (b) and NVP||Na/NST full cells (c). d) The long‐term cycling stability of the NVP||Na/NST and NVP||bare Na full cells at 5C.

To explore the practical application of the Na/NST electrode in the SMBs, the electrochemical performance of the full cells assembled the Na/NST anode with commercial NVP (Figure S13, Supporting Information) cathode (NVP||Na/NST) was examined, as exhibited in **Figure** **6**. As expected, compared with the NVP||bare Na, the NVP||Na/NST full cell shows a superior rate performance. Specially, it can stably cycle during the entire rate performance testing, and still retain an excellent reversible capacity of 65 mAh g^−1^ even at 50C. However, the NVP||bare Na full cell can only stably cycle at current densities below 10C, and is completely failed at 20C. Moreover, the charge–discharge curves of both full cells at various current densities from 1 to 50C are provided in the Figure 6b,c. The NVP||Na/NST full cell always possesses a smaller polarization voltage compared with the NVP||bare Na, indicating a significantly improved ionic transport kinetics. Furthermore, EIS was obtained after cycling, as presented in Figure S14 (Supporting Information). The NVP||Na/NST full cell possesses a considerably lower EIS value of 336 Ω than the NVP||bare Na full cell (1114 Ω), which further confirms the enhanced diffusion kinetics in the NVP||Na/NST full cell. And, the NVP||Na/NST full cell exhibits a superior cycle stability at 5C (Figures 6d and S15, Supporting Information). Even after 1000 cycles, it still retains a superior reversible discharge capacity of 90 mAh g^−1^ (corresponding to 88% of the initial capacity) and high Coulombic efficiency (CE) (over 99%). By contrast, a visibly capacity fading with a fluctuant capacity and unstable CE is observed in the NVP||bare Na full cell after 300 cycles (Figure S16, Supporting Information), implying the instable SEI on the bare Na electrode. This superior electrochemical performance of the Na/NST electrode suggests promising applications of the artificial interlayer strategy in SMBs.

## 3 Conclusion

An artificial SEI interlayer consisting of Na–Sn alloy, Sn, and Na_2_Te has been successfully fabricated on the surface of the Na substrate via SnTe powders pretreatment. The artificial SEI interlayer presents high sodiophilicity for rapid Na‐ion adsorption. And the artificial interlayer possesses low *E*_a_ of 40.9 kJ mol^−1^ and high exchange current density of 0.91 mA cm^−2^, which promote the Na‐ion fast transportation, achieving homogeneous Na plating. Moreover, the Na/NST electrode displays a satisfactory Young's modulus of 5.3 GPa, which effectively suppresses Na dendrite growth during the plating/stripping cycling. Owing to these merits of the artificial SEI interlayer, the symmetrical Na/NST cell displays a superior cycling life span over 1390 h at 1 mA cm^−2^ with 1 mAh cm^−2^ in carbonate‐based electrolyte. Furthermore, the NVP||Na/NST full cell retains a superior reversible capacity of 65 mAh g^−1^ even at 50C, and it also shows an excellent cycling stability (over 1000 cycles) at 5C with a high‐capacity retention of 88%. The design and fabrication of artificial interlayer on Na anode in our work is easy to scale‐up and feasible for practical application of SMBs.

## Conflict of Interest

The authors declare no conflict of interest.

## Supporting information

Supplementary Material

## Data Availability

The data that support the findings of this study are available from the corresponding author upon reasonable request.
