# Supplementary material for: Robust Artificial Interlayer with High Ionic Conductivity and Mechanical Strength toward Long‐Life Na‐Metal Batteries
Source: Small Sci. 2023 Jun 7;3(7):2300038. doi: 10.1002/smsc.202300038 (PMC11936041; doi:10.1002/smsc.202300038)
Supplement: Supplementary file 1 — Supplementary Material [file SMSC-3-2300038-s001.pdf]

Supporting Information

Robust Artificial Interlayer with High Ionic Conductivity and Mechanical Strength Towards Long-Life Na Metal Battery

Xianming Xia, Kaizhi Chen, Shitan Xu, Yu Yao, Lin Liu, Chen Xu, Xianhong Rui, and Yan Yu**

X. Xia, K. Chen, S. Xu, Dr. L. Liu, Prof. X. Rui
Guangdong Provincial Key Laboratory on Functional Soft Condensed Matter
School of Materials and Energy
Guangdong University of Technology
Guangzhou 510006, China
E-mail: xhrui@gdut.edu.cn

Dr. Y. Yao, Prof. Y. Yu
Hefei National Research Center for Physical Sciences at the Microscale
Department of Materials Science and Engineering
CAS Key Laboratory of Materials for Energy Conversion
University of Science and Technology of China
Hefei, Anhui 230026, China
E-mail: yanyumse@ustc.edu.cn

Dr. C. Xu
Academy for Advanced Interdisciplinary Studies
Southern University of Science and Technology
Shenzhen 518055, China

Experimental Section

Preparation of the Na/SnTe Electrodes:

The artificial SEI interlayer was constructed via a simple SnTe powders pretreatment. Typically, the commercial SnTe powders (Aladdin, 99.9%) was painted onto the fresh Na foil evenly. Then, the Na foil was held overnight. After that, the excess SnTe powders was cleaned away with a brush. Finally, the Na foil modified by SnTe was cut into Φ10 mm electrodes for coin-cell testing. The whole process was carried out inside the glove box filled with Ar gas (O_2 and H_2O < 0.1 ppm).

Materials Characterization:

The morphologies and elements distribution of artificial SEI interlayer were characterized by field emission scanning electron microscopy (SEM, JEOL, JSM-6360LA). The phase structures and compositions of artificial interlayer before and after cycling were investigated by X-ray diffraction (XRD, Bruker with a Cu Kα radiation), Cryogenic transmission electron microscopy (Cryo-TEM, JEOL JEM-F200) and X-ray photoelectron spectroscopy (XPS, Thermo Fisher ESCALAB 250Xi).

Electrochemical Characterizations:

The electrochemical performances were tested in 2032 coin-type cells at room temperature. The electrolyte of symmetrical cells and full cells was 1.0 M $NaPF_6$ in EC: DEC = 1:1 (volume ratio) with 5% FEC as the additive. The dosage of electrolyte used in all cells was 200 μL. The separator of the symmetrical and full cells was Whatman GF/D glass fiber. And, the $Na_3V_2(PO_4)_3$ (NVP) cathode was fabricated by coating the slurry (a mixture of 80 wt% commercial NVP (purchased from *Guangdong Canrd New Energy Technology Co., Ltd.*), 10 wt% CNT, and 10 wt% PVDF) on Al foil and then dried under vacuum at 60 °C overnight. The mass loading of NVP was about 2.0-3.0 mg cm^{-2}. The electrochemical performance of all cells was tested using the Neware testing system. The electrochemical impedance spectroscopy (EIS, 0.01-10^5 Hz) was obtained by CHI660 workstation.

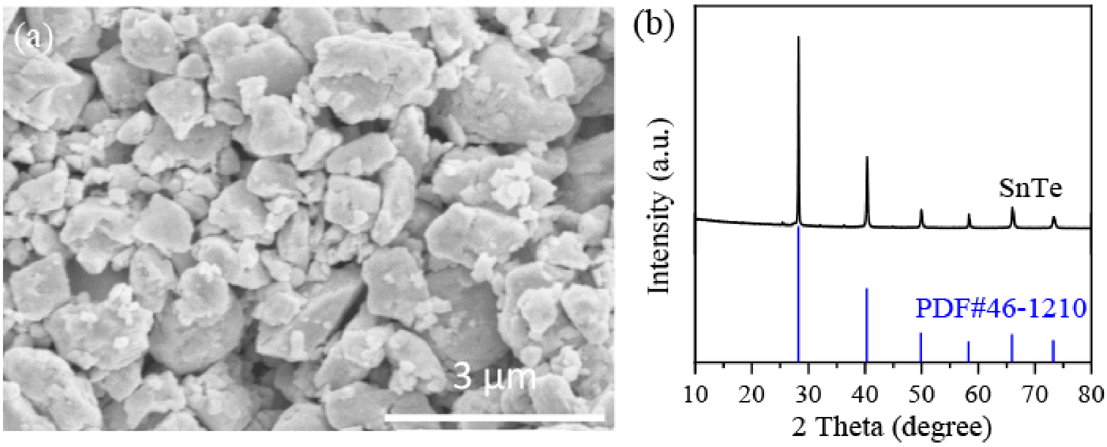

Figure S1. (a) SEM image and (b) XRD pattern of SnTe powders.

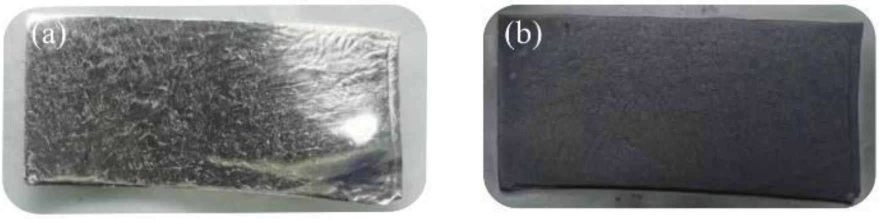

Figure S2. Optical images of (a) bare Na and (b) Na foil after painting SnTe powders.

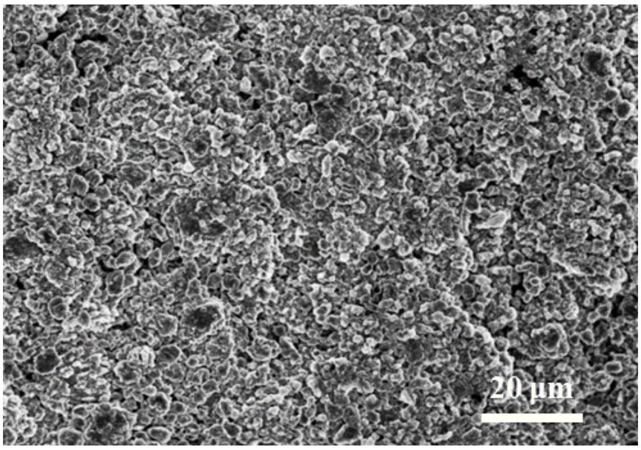

Figure S3. The SEM image of Na/NST electrode.

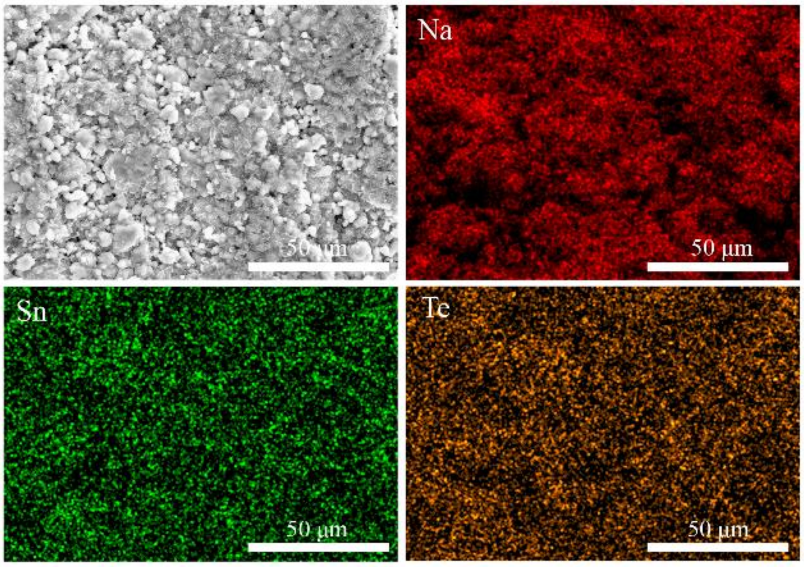

Figure S4. The surface morphology and SEM EDS elemental mapping of Na/NST electrode.

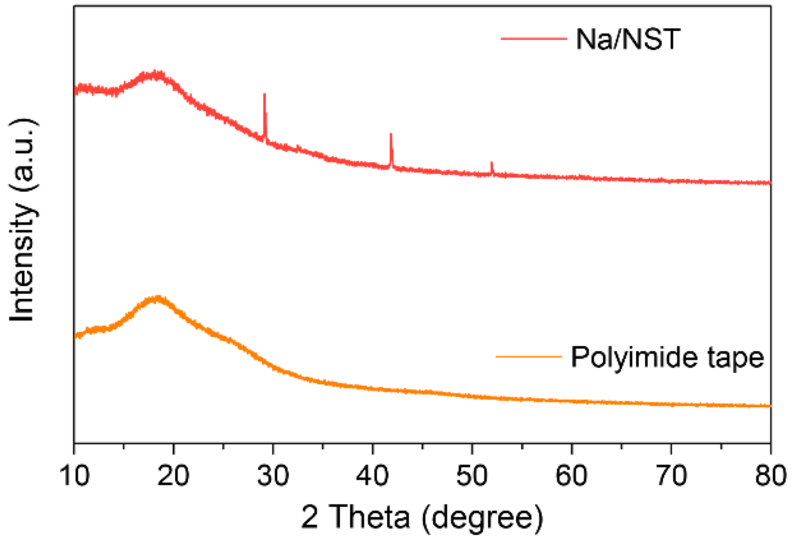

Figure S5. The XRD patterns of Na/NST and polyimide tape.

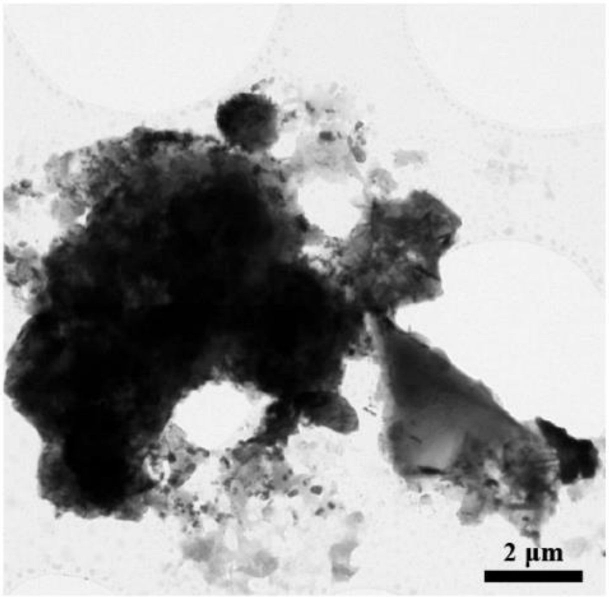

Figure S6. The Cryo-TEM image of the artificial NST interlayer.

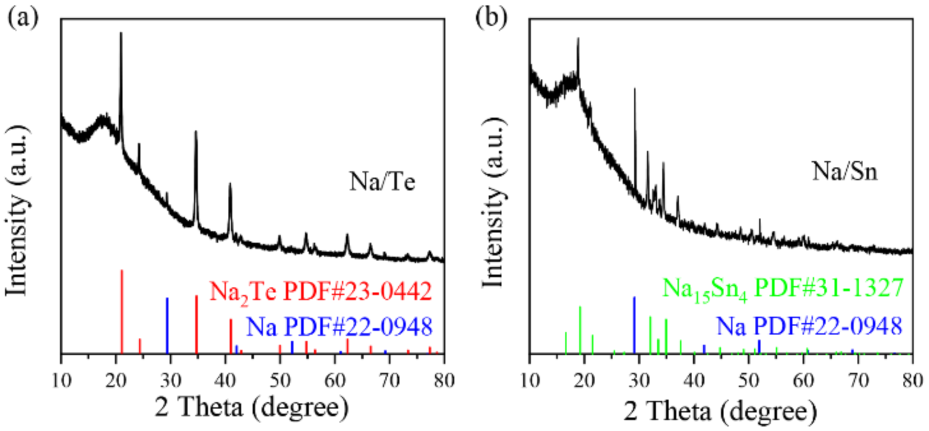

Figure S7. The XRD patterns of (a) Na/Te and (b) Na/Sn electrodes.

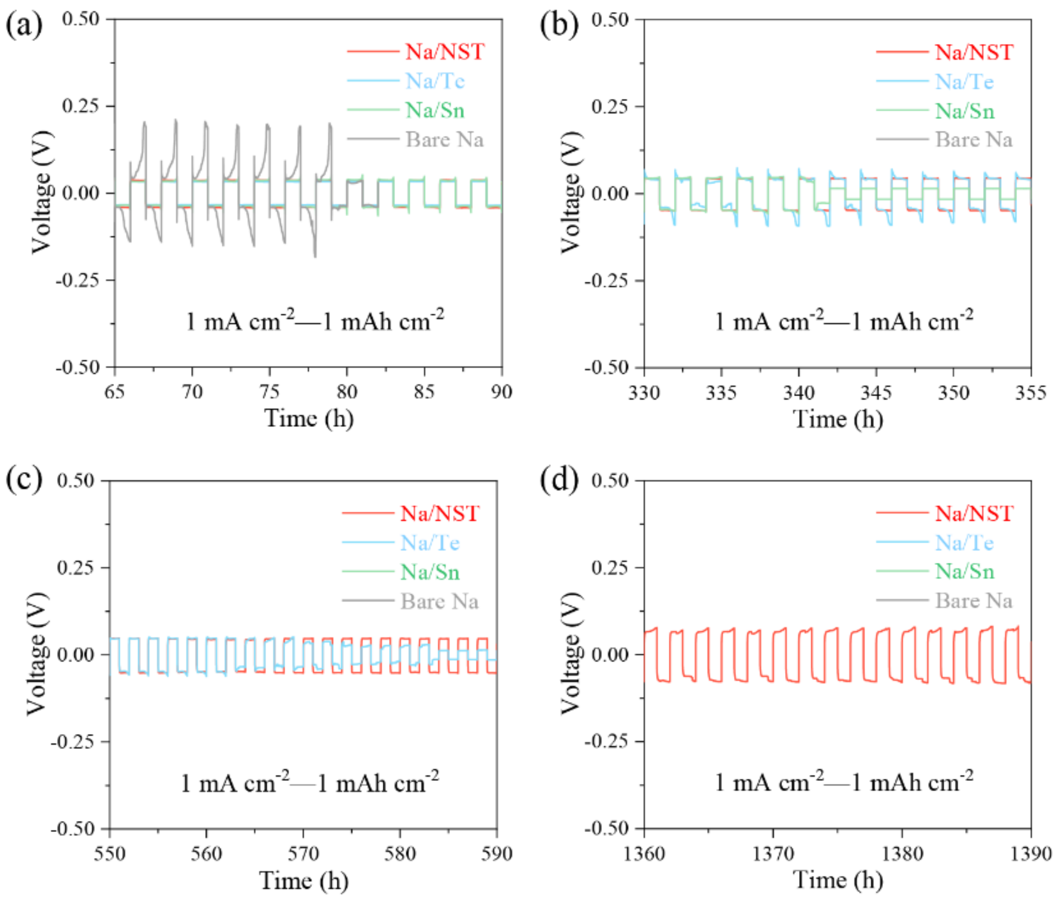

Figure S8. The detailed plating/stripping curves at (a) 65-90 h, (b) 330-355 h, (c) 550-590 h and (d) 1360-1390 h of the symmetrical bare Na, Na/Sn, Na/Te and Na/NST cells at 1 mA cm^{-2} with a fixed capacity of 1 mAh cm^{-2}.

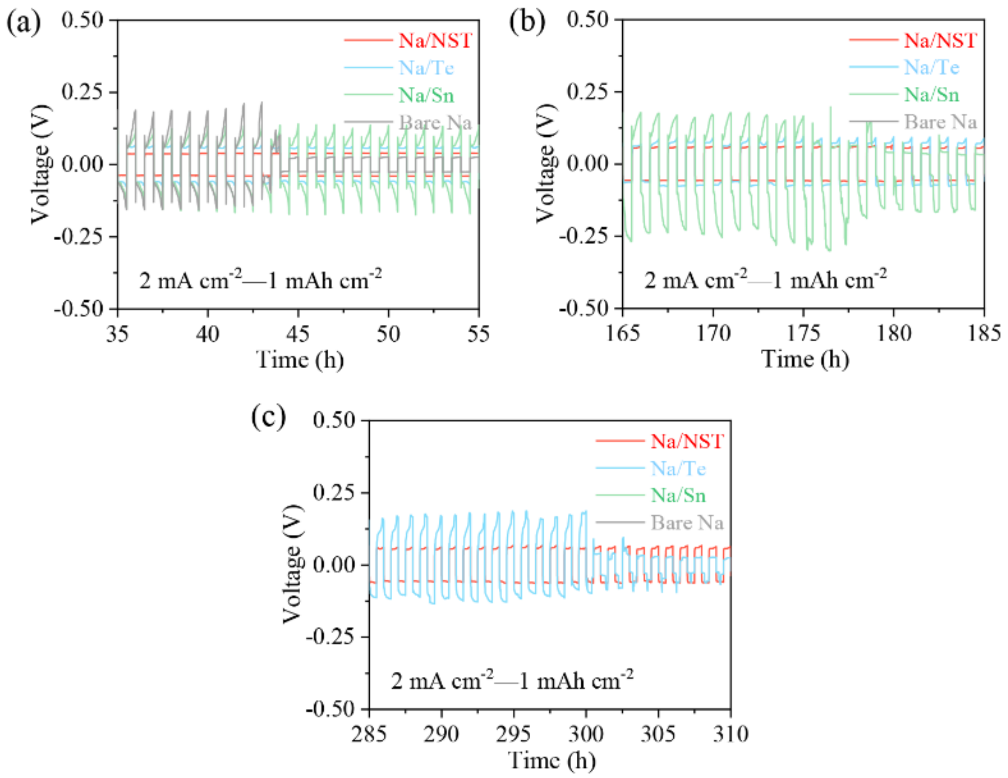

Figure S9. The detailed plating/stripping curves at (a) 35-55 h, (b) 165-185 h and (c) 285-310 h of the symmetrical bare Na, Na/Sn, Na/Te and Na/NST cells at 2 mA cm^{-2} with a fixed capacity of 1 mAh cm^{-2}.

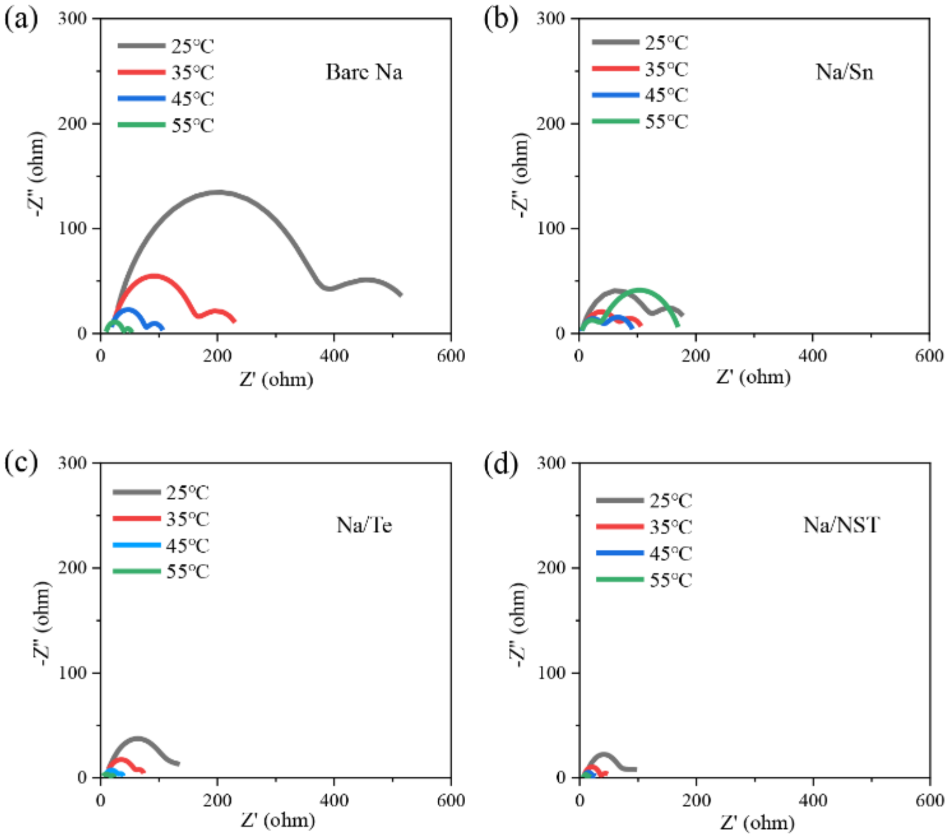

Figure S10. The EIS curves of symmetric (a) bare Na, (b) Na/Sn, (c) Na/Te and (d) Na/NST cells after 10 cycles at different temperatures.

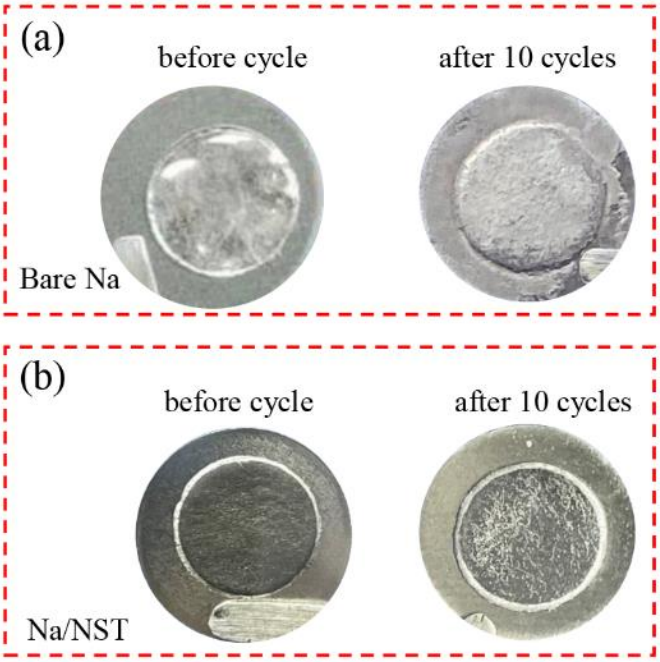

Figure S11. The optical images of (a) bare Na and (b) Na/NST electrodes before and after cycling at 1 mA cm^{-2} with 1 mAh cm^{-2}.

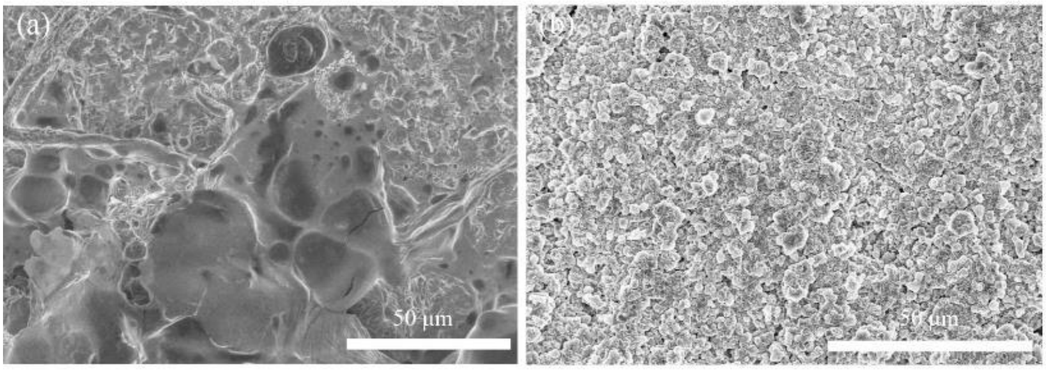

Figure S12. The SEM images of (a) bare Na and (b) Na/NST electrodes after 10 cycles at 1 mA cm^{-2} with 1 mAh cm^{-2}.

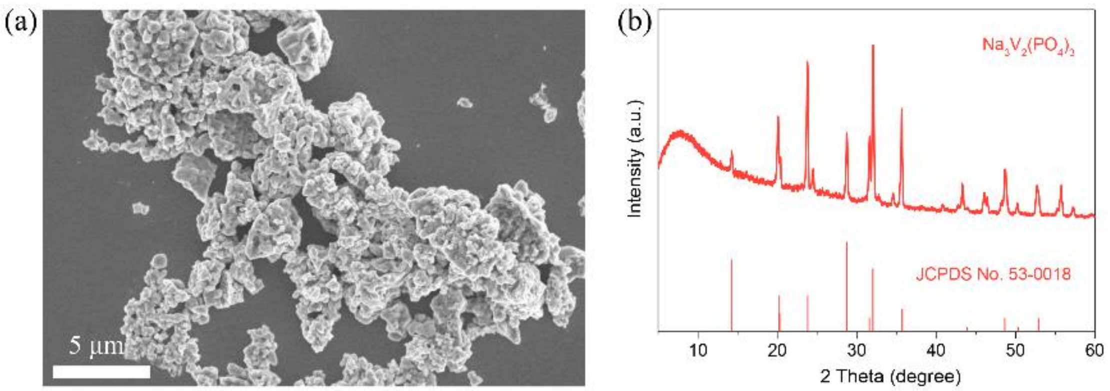

Figure S13. (a) SEM image and (b) XRD pattern of the NVP.

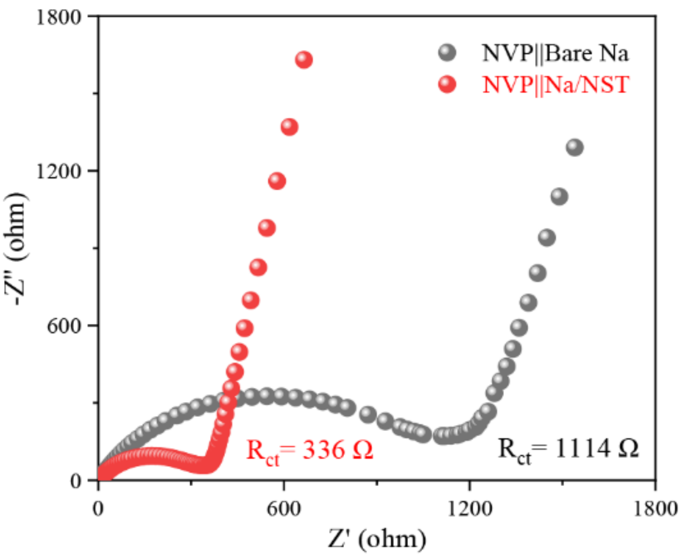

Figure S14. The EIS curves of full cells with different anodes after cycling at 5C for 100 cycles.

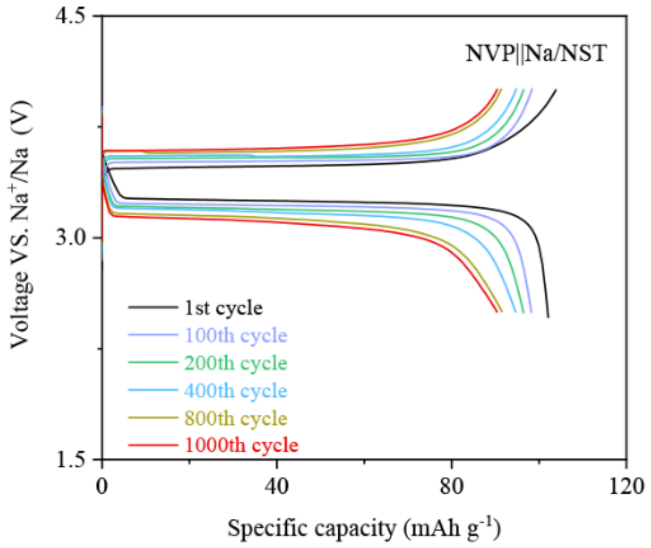

Figure S15. The charge/discharge curves of NVP||Na/NST full cell during the 1st, 100th, 200th, 400th, 800th and 1000th cycles.

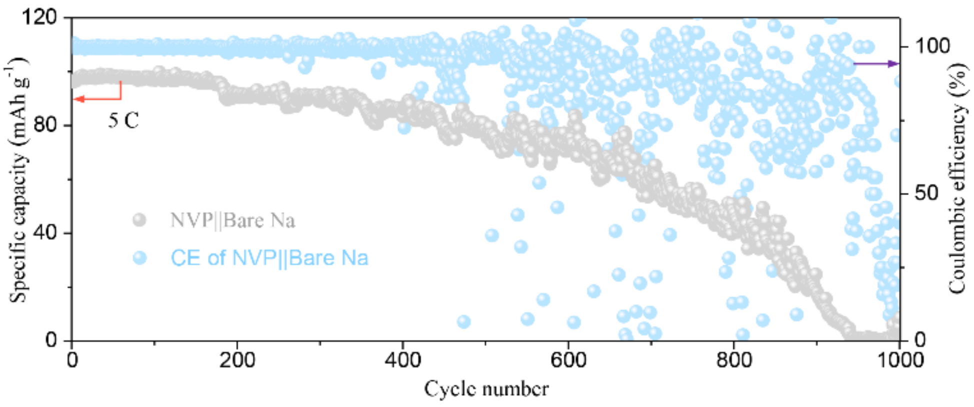

Figure S16. The long-term cycling stability and the CE of the NVP||bare Na full cell at 5 C.

Table S1 A comparison of the cycling stability of our Na/NST with previously advanced Na anodes.

Na metal Anode	Electrolyte	Fixed capacity	Current density	Lifespan	Ref.
AES-G	1 M $NaClO_4$ in EC:PC with 5% FEC	1 mAh cm^{-2}	2 mA cm^{-2}	100 h	[1]
Na-Bi	1 M $NaPF_6$ in EC:DEC:DMC with 5% FEC	1 mAh cm^{-2}	1 mA cm^{-2}	500 h	[2]
Na@Na_2Se/V	1 M $NaClO_4$ in EC:DEC with 5% FEC	1mAh cm^{-2}	1mA cm^{-2}	625 h	[3]
NGAL-Na	1 M $NaClO_4$ in EC:DEC with 5% FEC	1mAh cm^{-2}	1mA cm^{-2}	642 h	[4]
NaBrP	1 M $NaPF_6$ in EC:DEC with 5% FEC	1 mAh cm^{-2}	1 mA cm^{-2}	700 h	[5]
Na@Na_2Te	1 M $NaClO_4$ in EC:DEC with 5% FEC	1mAh cm^{-2}	1mA cm^{-2}	700 h	[6]
Na_3P@Na	1 M NaTFSI in FEC/EMC	1 mAh cm^{-2}	1 mA cm^{-2}	780 h	[7]
NaF/Co/Na	1 M $NaClO_4$ in EC:DEC with 5% FEC	1mAh cm^{-2}	1mA cm^{-2}	1000 h	[8]
Na/NST	1 M $NaPF_6$ in EC:DEC with 5% FEC	1 mAh cm^{-2}<br>1 mAh cm^{-2}	1 mA cm^{-2}<br>2 mA cm^{-2}	1390 h<br>380 h	This work

References

[1] W. Liu, P. Li, W. Wang, D. Zhu, Y. Chen, S. Pen, E. Paek, D. Mitlin, *ACS Nano* **2018,** *12*, 12255.

[2] G. Yang, N. Li, C. Sun, *ACS Applied Energy Materials* **2020,** *3*, 12607.

[3] X. Xia, S. Xu, F. Tang, Y. Yao, L. Wang, L. Liu, S. He, Y. Yang, W. Sun, C. Xu, Y. Feng, H. Pan, X. Rui, Y. Yu, *Adv. Mater.* **2022**, e2209511.

[4] X. Lv, F. Tang, Y. Yao, C. Xu, D. Chen, L. Liu, Y. Feng, X. Rui, Y. Yu, *SusMat* **2022,** *2*, 699.

[5] Z. Luo, S. Tao, Y. Tian, L. Xu, Y. Wang, X. Cao, Y. Wang, W. Deng, G. Zou, H. Liu, H. Hou, X. Ji, *Nano Energy* **2022,** *97*, 107203.

[6] H. Yang, F. He, M. Li, F. Huang, Z. Chen, P. Shi, F. Liu, Y. Jiang, L. He, M. Gu, Y. Yu, *Adv. Mater.* **2021,** *33*, e2106353.

[7] P. Shi, S. Zhang, G. Lu, L. Wang, Y. Jiang, F. Liu, Y. Yao, H. Yang, M. Ma, S. Ye, X. Tao, Y. Feng, X. Wu, X. Rui, Y. Yu, *Advanced Energy Materials* **2020,** *11*, 2003381.

[8] X. Zhou, F. Liu, Y. Wang, Y. Yao, Y. Shao, X. Rui, F. Wu, Y. Yu, *Advanced Energy Materials* **2022,** *12*, 2202323.
